# Direct comparison of coronary bare metal vs. drug-eluting stents: same platform, different mechanics?

**DOI:** 10.1186/s40001-017-0300-y

**Published:** 2018-01-08

**Authors:** Wolfram Schmidt, Peter Lanzer, Peter Behrens, Christoph Brandt-Wunderlich, Alper Öner, Hüseyin Ince, Klaus-Peter Schmitz, Niels Grabow

**Affiliations:** 1 Institute for Biomedical Engineering, University Medical Center Rostock, Friedrich-Barnewitz-Strasse 4, D-18119, Rostock-Warnemuende, Germany; 2 Center for Internal Medicine, Health Center Bitterfeld/Wolfen gGmbH, Academic Teaching Hospital of the Martin-Luther-University Halle-Wittenberg, Bitterfeld-Wolfen, Germany; 3 Institute for ImplantTechnology and Biomaterials–IIB e.V., Associated Institute of the University of Rostock, Rostock-Warnemuende, Germany; 4 Department for Cardiology, Center for Internal Medicine, University Medical Center Rostock, Rostock, Germany

**Keywords:** Interventional cardiology, Bare metal stents, Drug-eluting stents, Biomechanics

## Abstract

**Background:**

Drug-eluting stents (DES) compared to bare metal stents (BMS) have shown superior clinical performance, but are considered less suitable in complex cases. Most studies do not distinguish between DES and BMS with respect to their mechanical performance. The objective was to obtain mechanical parameters for direct comparison of BMS and DES.

**Methods:**

In vitro bench tests evaluated crimped stent profiles, crossability in stenosis models, elastic recoil, bending stiffness (crimped and expanded), and scaffolding properties. The study included five pairs of BMS and DES each with the same stent platforms (all *n* = 5; PRO-Kinetic Energy, Orsiro: BIOTRONIK AG, Bülach, Switzerland; MULTI-LINK 8, XIENCE Xpedition: Abbott Vascular, Temecula, CA; REBEL Monorail, Promus PREMIER, Boston Scientific, Marlborough, MA; Integrity, Resolute Integrity, Medtronic, Minneapolis, MN; Kaname, Ultimaster: Terumo Corporation, Tokyo, Japan). Statistical analysis used pooled variance *t* tests for pairwise comparison of BMS with DES.

**Results:**

Crimped profiles in BMS groups ranged from 0.97 ± 0.01 mm (PRO-Kinetic Energy) to 1.13 ± 0.01 mm (Kaname) and in DES groups from 1.02 ± 0.01 mm (Orsiro) to 1.13 ± 0.01 mm (Ultimaster). Crossability was best for low profile stent systems. Elastic recoil ranged from 4.07 ± 0.22% (Orsiro) to 5.87 ± 0.54% (REBEL Monorail) including both BMS and DES. The bending stiffness of crimped and expanded stents showed no systematic differences between BMS and DES neither did the scaffolding.

**Conclusions:**

Based on in vitro measurements BMS appear superior to DES in some aspects of mechanical performance, yet the differences are small and not class uniform. The data provide assistance in selecting the optimal system for treatment and assessment of new generations of bioresorbable scaffolds.

*Trial registration*: not applicable

## Background

Depending on case selection, about one-third of percutaneous coronary intervention (PCI) cases can be considered technically complex due to a difficult access to or difficult crossing of the target lesion. The technical adversity is characterized by tortuous pathways, sharp angled and up-sloping take offs, highly calcified ostia, diffusely diseased small vessels, vessels with large soft or shaggy calcified plaques, irregular surface textures, small diffusely diseased target sites and high-grade stenoses. All these factors may potentially contribute to case complexity. While numerous strategies have been proposed to overcome these adversities, little has been reported concerning the selection of the optimum instrumentation. Specifically, the mechanical properties of the most suitable stent delivery system still remain to be defined.

The superior clinical performance of drug-eluting stents (DES) compared to bare metal stents (BMS) has been widely documented and confirmed. Thus, economic considerations aside, based on clinical performance DES, should be selected in most clinical scenarios [1]. On the other hand, the added benefit of DES in some subsets of lesions still remains a subject of scientific debates [2]. However, while being mostly discussed based on clinical empiricism, differences between BMS and DES with respect to their mechanical properties have been rarely considered.

It is widely believed that the potential advantages of BMS compared to DES may be better mechanical properties, such as low profile, better trackability and crossability. However, to our knowledge, comparative BMS and DES head-on measurements have not been performed previously. If true, BMS would appear to represent first choice instrumentation in bail-out indications. Here, we report in vitro measurements of crossing profiles and flexibility of representative DES and BMS stent delivery systems (SDS) allowing objective estimates of expected behaviour in vivo. In addition, measurements of the cell sizes of stents inflated to nominal pressures have also been performed to allow estimates of scaffolding efficacy.

Definition of mechanical properties and their comparison in current generation of BMS and DES also appears critical for the future generations of bioresorbable scaffolds (BRS) to establish reproducible target values for required performance.

## Methods

**Fig. 1:**
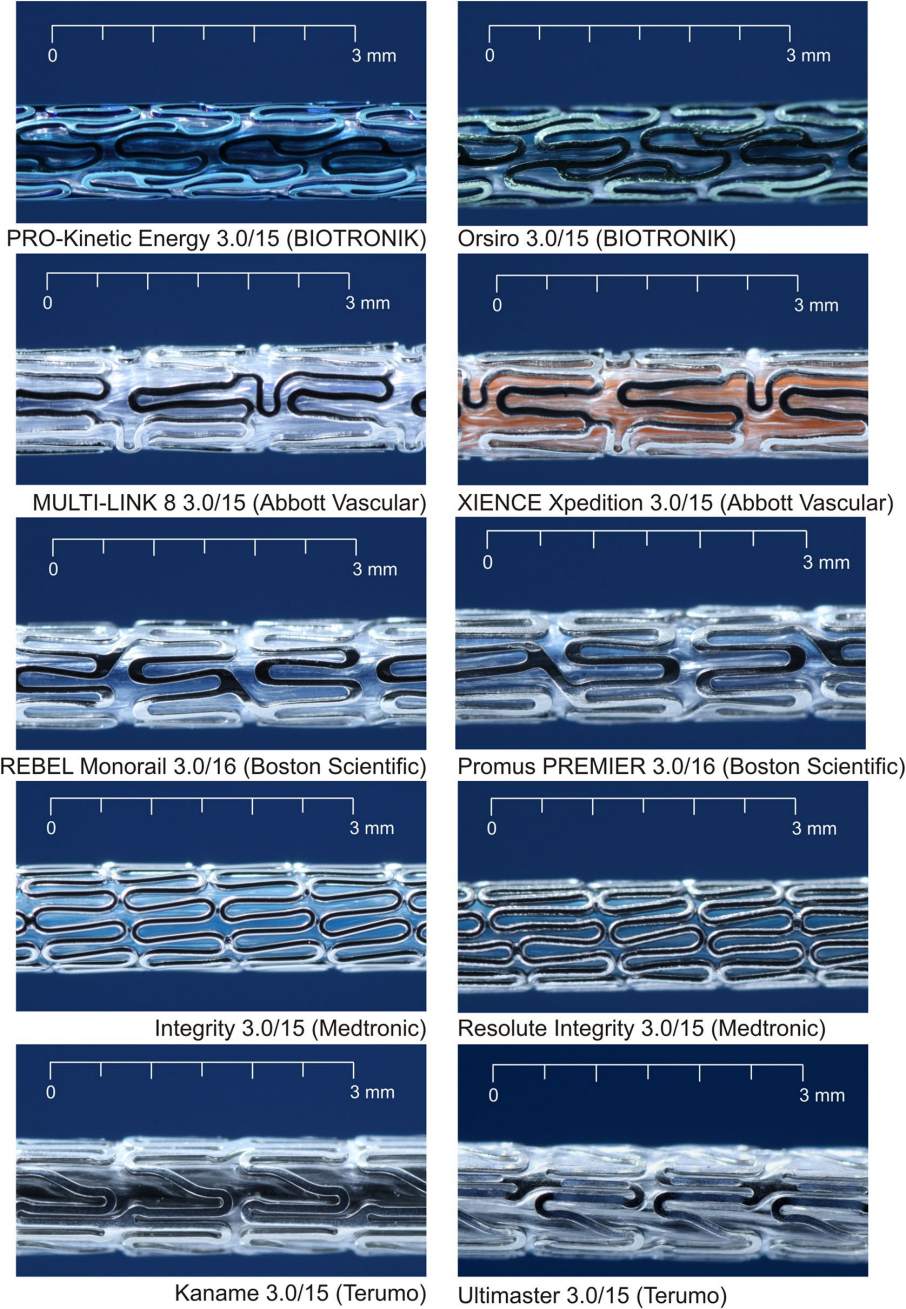
Investigated BMS (left) and DES (right) based on same stent platforms, respectively

Five different BMS and corresponding DES systems with the same stent platforms were investigated. Measurements were performed on five stents in each group. Thus, in total 50 stents were measured. The following BMS/DES stent pairs were investigated (Fig. 1): PRO-Kinetic Energy/Orsiro (BIOTRONIK AG, Bülach, Switzerland), MULTI-LINK 8/XIENCE Xpedition (Abbott Vascular, Temecula, CA), REBEL Monorail/Promus PREMIER (Boston Scientific, Marlborough, MA), Integrity/Resolute Integrity (Medtronic, Minneapolis, MN), Kaname/Ultimaster (Terumo Corporation, Tokyo, Japan).

Manufacturer information on stent material, strut dimensions and coatings is summarized in Table 1. Most stents consist of a cobalt-chromium alloy (L-605). Exceptions include the Integrity and Resolute Integrity that are made from a CoNi-base alloy (MP35N) and the REBEL and Promus PREMIER stents that are made from a platinum-chromium alloy (PtCr).

**Table 1:** Material parameters of investigated BMS (italics) and DES (roman)

Stent type	Stent material	Strut thickness (µm)	Coating	Drug	References
*PRO-Kinetic Energy*	*CoCr L-605*	*60*	*proBIO amorphous silicon carbide coating*	*–*	[3]
Orsiro	CoCr L-605	60	proBIO amorphous silicon carbide coating/BIOlute bioresorbable Poly-l-Lactide	Sirolimus (1.4 µg/mm^2^)	[12]
*MULTI-LINK 8*	*CoCr L-605*	*81*	*–*	*–*	[4, 13]
XIENCE Xpedition	CoCr L-605	89 (including coating)	Non-erodible polymer poly *n*-butyl methacrylate (PBMA)/vinylidene fluoride-co-hexafluoropropylene (PVDF-HFP)	Everolimus (100 µg/cm^2^)	[11, 14]
*REBEL Monorail*	*PtCr*	*81*	*–*	*–*	[5]
Promus PREMIER	PtCr	81	Non-erodible polymer poly *n*-butyl methacrylate (PBMA)/vinylidene fluoride-co-hexafluoropropylene (PVDF-HFP)	Everolimus (100 µg/cm^2^)	[15]
*Integrity*	*CoNi MP35* *N*	*90*	*–*	*–*	[6–8]
Resolute integrity	CoNi MP35 N	90	BioLinx (PVP, C10, and C19)	Zotarolimus (1.6 µg/mm^2^)	[7, 16]
*Kaname*	*CoCr L-605*	*80*	*–*	*–*	[9, 10]
Ultimaster	CoCr L-605	80	Poly (dl-lactide-co-caprolactone)	Sirolimus (3.9 μg/mm stent length)	[17]

The strut thickness varies from 60 µm (PRO-Kinetic Energy, Orsiro) to 90 µm (Integrity, Resolute Integrity). All studied DES use a Limus derivate as drug to control restenosis (Sirolimus, Everolimus, and Zotarolimus). Differences exist with respect to the drug carrier matrix polymers and the drug load, all listed in the product literature (Table 1). In general, the mechanical strength of the coating material and the coating thickness both are small compared to the metal bulk material of the stent, and a mechanical impact is not necessarily expected. However, the manufacturing process of securing the drug and polymer on the stent might also alter mechanical stent properties and should be of interest. Additionally, despite the comparable stent platforms of BMS and DES, differences in material and construction of the delivery catheters may be different. These details are not reported by manufacturers, but affect crossability or bending stiffness of the complete stent systems.

### Crimped profile

The crimped profile was measured to obtain a simple but relevant parameter to characterize the geometry of the distal balloon/stent section. The crimpled profile has been determined using a 2-axis laser scanner (ODAC 64 XY, Zumbach Electronic, Switzerland), which is part of a specific setup for stent testing [18]. The system measures the diameter of the catheter and mounted stent in two perpendicular directions (*x*, *y*). The profile at the measurement plane is summarized as root mean square (RMS) value. These diameter measurements were taken in steps of 0.2 mm along the distal part of the stent systems. The mean profile of the crimped stent was derived by averaging of all measured values in the stent region.

### Crossability

The crossability as a functional parameter to assess the ease of passing a narrowed lesion has been determined using a setup described previously [19, 20]. Briefly, a simulated vessel curvature was added by a stenotic lesion model at the end. The lesion model was fixed at a load cell (type 3482, burster, measurement range ± 2 N, *u*_95_ = ± 0.034 N). The lesion model was placed in 37 °C heated water. The stent system to be tested was fixed with its proximal grip at a linear motor drive. A second load cell was used to check maximum push force during automatic advancement of the system. The distal reaction force was recorded during passage of the stenotic lesion model and the average was calculated as the measure of crossability. The test was repeated three times and the resulting forces were averaged.

The lesion model is characterized by a narrowing ranging from 2.5 to 1.1 mm, corresponding to a diameter reduction of 56% (cross section area reduction 81%). The lesion diameter was increased to 1.2 mm (lesion diameter reduction by 52%, area reduction 77%) in case the tested device could not pass without exceptional high reactive frictional forces. Thus, the systems tested at 1.1 mm stenosis had a better crossability than those tested at 1.2 mm narrowing.

### Bending stiffness

The bending stiffness is a measure of structural resistance to bending deformation; it is the reciprocal of flexibility. The bending stiffness of the balloon section with mounted stent was measured using an experimental setup where the test sample is fixed and deflected at a distance l (free bending length) by the deflection f. The resulting bending force *F* (load cell PW4MC3/300G-1, Hottinger Baldwin, measurement range ± 3 N, accuracy ± 0.48 mN) is measured for increasing but small deflections. The force–distance curves describe the elastic behaviour of the test objects with respect to bending. The bending stiffness EI is calculated taking the mean value of *F*/*f* calculated by linear regression from the whole force–distance curve (three measurements per direction). Considering possible asymmetric structures of the test samples, the bending stiffness is measured in five directions around the circumference and subsequently averaged [18].

The bending stiffness was also measured for expanded stents where a low stiffness is assumed to enable easy adaptation to vessel curvatures.

### Elastic recoil

Stent expansion behaviour, in particular the elastic recoil, was investigated in vitro by applying the balloon pressure through a computer-controlled piston pump (Nemesys, Cetoni) and measuring the stent outer diameter as a function of balloon pressure. For this purpose, the 2-axis laser scanner was used as mentioned above [18]. The stents were expanded to nominal pressure (NP) as provided by the manufacturer’s instructions for use (IFU). Then, the balloon was deflated and the stent profile was measured again. The elastic recoil was derived from both diameters: the diameter *d*_max_ at NP and the reduced diameter *d*_0_ after recoil at zero pressure.

### Scaffolding

The expanded stent structures were assessed with respect to their scaffolding potential. The parameter was the maximum circular opening at the individual stents. This approach has also been described for assessment of side branch accessibility or cell opening in context of bifurcation stenting [21]. Imaging of stents in their expanded state (nominal conditions: expanded to NP) was conducted using an SZX16 microscope (Olympus). Maximum circles were inserted and measured with the calibrated image analysis software Stream (Olympus). Three circles per stent were measured.

### Statistics

Pooled variance *t* test for independent samples (SPSS Statistics 22, IBM) was used for statistical analysis. The significance level was set as *p* < 0.05.

## Results

All numerical results are summarized in Table 2.

**Table 2:** Summary of results, comparison of BMS vs. DES

	*PRO-Kinetic Energy* *3.0/15*		Orsiro 3.0/15		*MULTI-LINK 8* *3.0/15*		XIENCE Xpedition 3.0/15		*REBEL Monorail* *3.0/16*		Promus PREMIER 3.0/16		*Integrity* *3.0/15*		Resolute Integrity 3.0/15		*Kaname* *3.0/15*		Ultimaster 3.0/15	
Outer diameter of crimped system (mm)	*0.97*	*± 0.01*	1.02	± 0.01	*1.09*	*± 0.01*	1.10	± 0.01	*1.05*	*± 0.01*	1.05	± 0.01	*1.07*	*± 0.01*	1.09	± 0.01	*1.13*	*± 0.01*	1.13	± 0.01
Crossability (N)																				
Stenosis 1.1 mm	*0.034*	*± 0.001*	0.142	± 0.017	*–*	^*a*^	0.258	± 0.036	*0.098*	*± 0.014*	0.287	± 0.045	*–*	^*a*^	–	^a^	*–*	^*a*^	–	^a^
Stenosis 1.2 mm					*0.089*	*±0.014*							*0.255*	*±0.011*	0.294	±0.044	*–*	^*a*^	0.409	^b^
Bending stiffness (Nmm^2^)																				
With crimped stent	*22.8*	*± 3.0*	34.3	± 3.2	*42.7*	*± 3.6*	38.2	± 2.3	*31.6*	*± 2.1*	30.5	± 2.8	*59.2*	*± 4.7*	98.7	± 8.6	*36.4*	*± 2.0*	33.3	± 2.2
Expanded stent	*8.9*	*± 1.4*	8.8	± 0.9	*9.6*	*± 1.4*	11.0	± 1.4	*3.6*	*± 0.9*	4.0	± 0.7	*7.5*	*± 0.1*	7.7	± 0.1	*4.3*	*± 0.3*	4.4	± 0.2
Elastic recoil (%)	*5.34*	*± 0.36*	4.07	± 0.22	*5.00*	*± 0.31*	5.12	± 0.19	*5.87*	*± 0.54*	5.15	± 0.71	*5.61*	*± 0.53*	5.70	± 0.26	*5.27*	*± 0.57*	5.19	± 0.18
Scaffolding, open cell diameter (mm)	*0.910*	*± 0.163*	0.958	± 0.058	*0.836*	*± 0.023*	1.008	± 0.024	*0.822*	*± 0.015*	0.820	± 0.035	*0.610*	*± 0.015*	0.855	± 0.055	*0.756*	*± 0.03*	0.751	± 0.041

^a^Not able to pass the stenosis model with specified diameter

^b^Only one system has passed the stenosis model with *d* = 1.2 mm

### Crimped profile

**Fig. 2:**
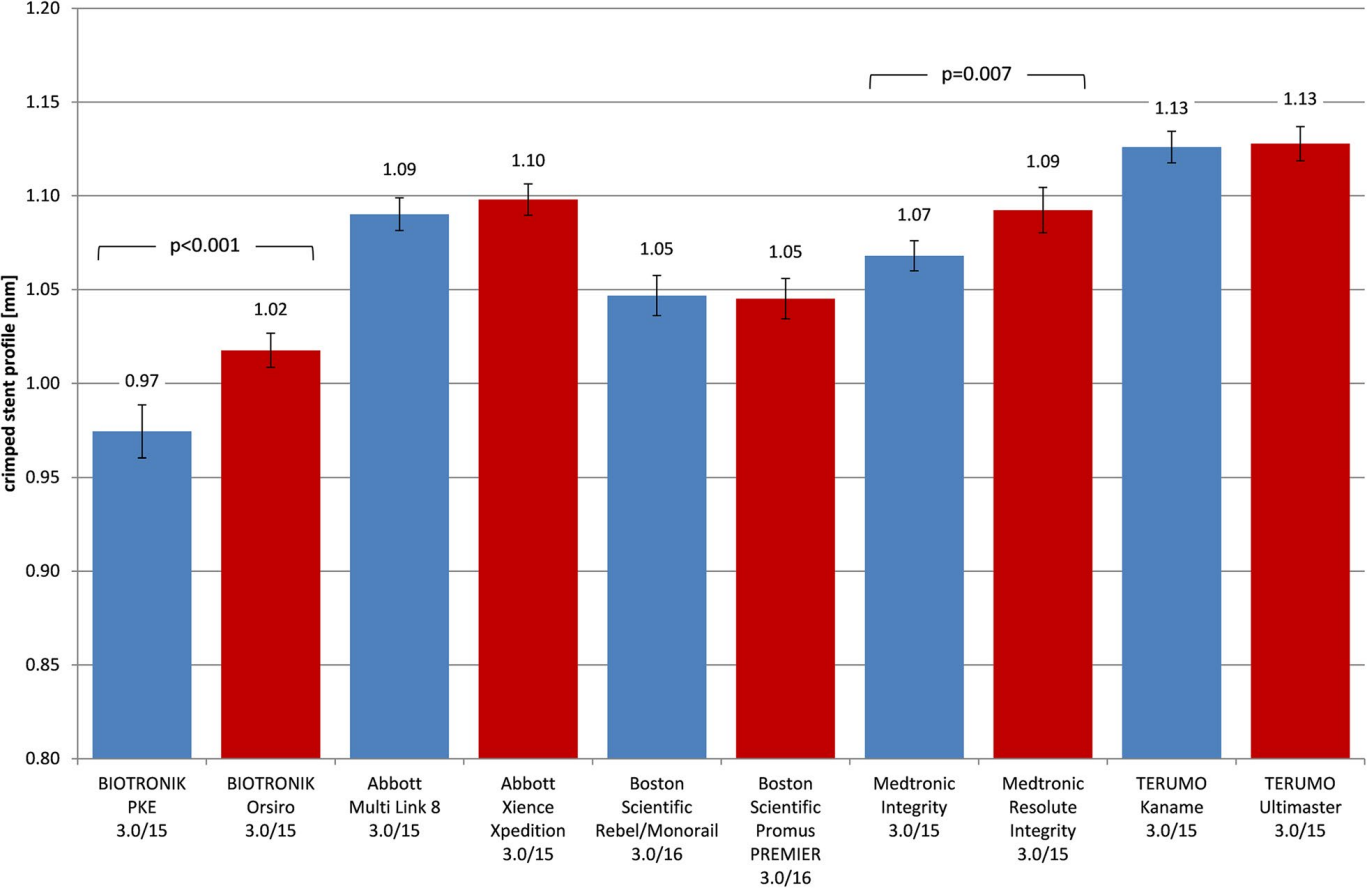
Comparison of profiles of crimped stents (BMS blue, DES red)

The profile of crimped stents was measured in the stent region on the balloon catheter (Fig. 2). The stent profiles of BMS ranged from 0.97 mm (PRO-Kinetic Energy) to 1.13 mm (Kaname). In the DES group, the profiles ranged from 1.02 mm (Orsiro) to 1.13 mm (Ultimaster). The standard deviation was low for all profile measurements (± 0.01 mm). The profiles of BMS were equal or smaller than DES of the same design; for the BIOTRONIK PRO-Kinetic Energy vs. Orsiro (*p* < 0.001) and Medtronic Integrity vs. Resolute Integrity, the differences were highly significant (*p* = 0.007). No DES had a smaller profile than the corresponding BMS.

### Crossability

**Fig. 3:**
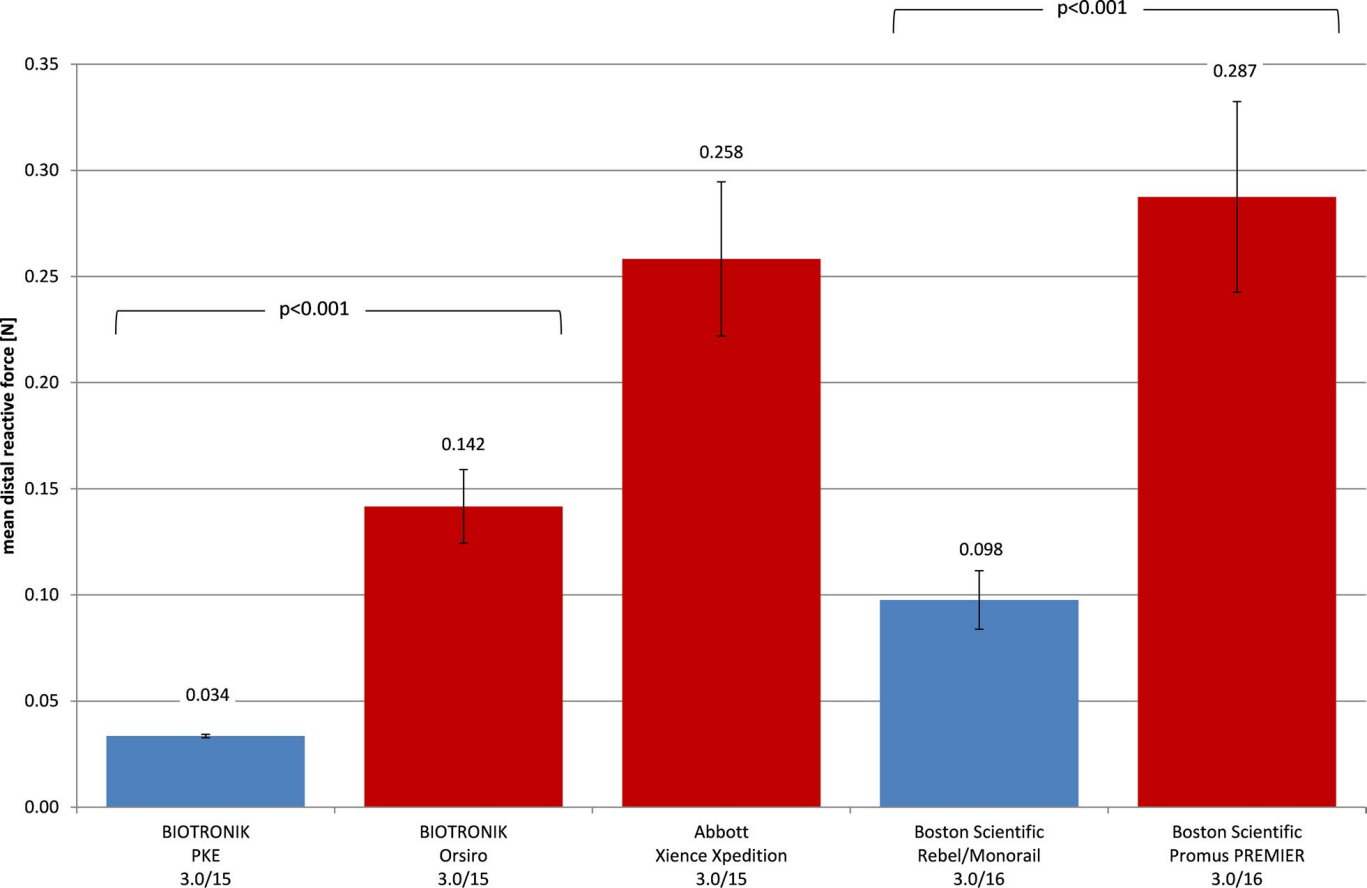
Mean distal forces $\overline{{F_{\text{dist}} }}$ of crossability tests (stenosis model *d* = 1.1) as a measure of crossability (with standard deviation, BMS blue, DES red)

Five stent system types (2 BMS and 3 DES) were able to pass a 1.1 mm stenosis model. The mean distal forces as the measure of crossability are compared in Fig. 3. The forces ranged from 0.034 ± 0.001 N (PRO-Kinetic Energy) to 0.287 ± 0.045 N (Promus PREMIER). Pairwise comparison of BMS vs. DES provided significant better crossability for BIOTRONIK PRO-Kinetic Energy vs. Orsiro (*p* < 0.001) and Boston Scientific REBEL/Monorail vs. Promus PREMIER (*p* < 0.001).

**Fig. 4:**
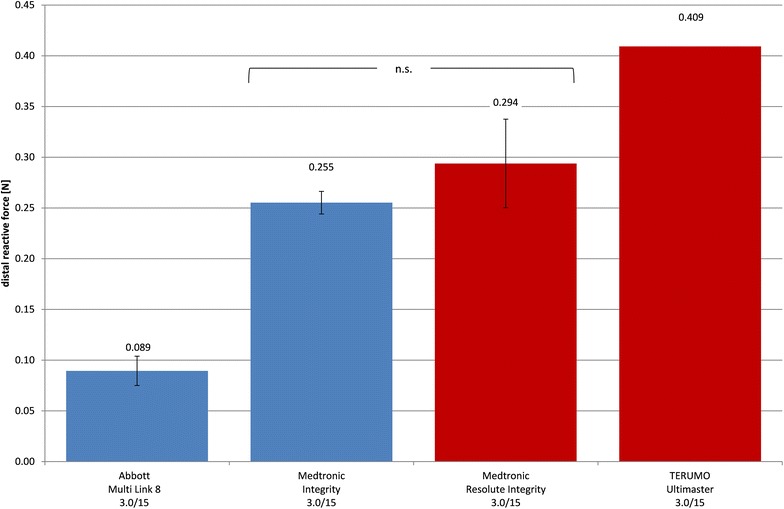
Mean distal forces $\overline{{F_{\text{dist}} }}$ of crossability tests (stenosis model *d* = 1.2) as a measure of crossability (with standard deviation, BMS blue, DES red)

All other stent systems had to be tested with a stenosis model of 1.2 mm diameter (Fig. 4) and thus have a lower crossability than stents shown in Fig. 3. The mean distal forces ranged from 0.089 ± 0.014 N (MULTI-LINK 8) to 0.409 N (Ultimaster). The differences in crossability of Medtronic Integrity and Resolute Integrity were not significant.

The BMS Kaname could not pass the 1.2 mm stenosis model.

### Bending stiffness of catheter/stent

**Fig. 5:**
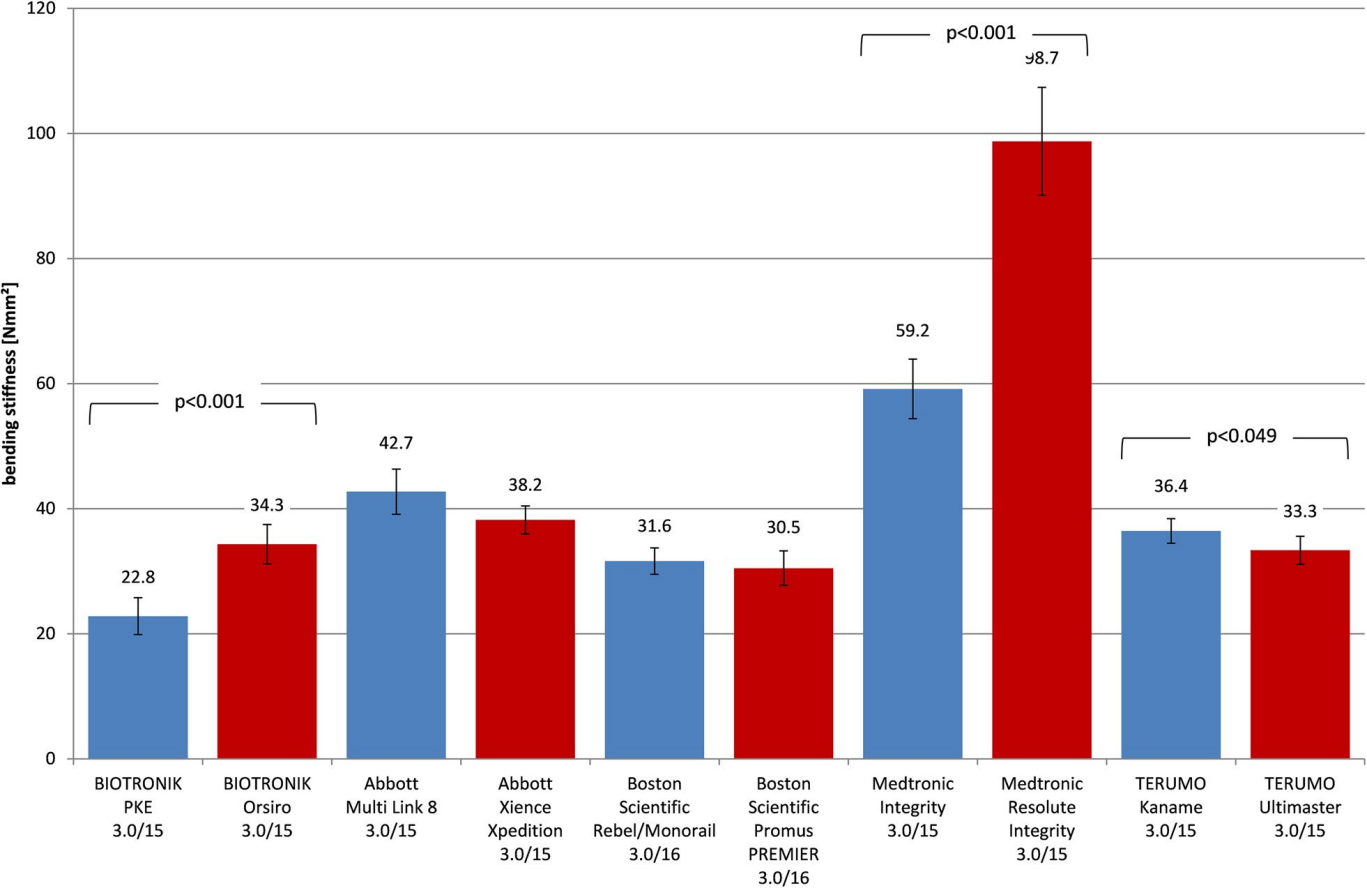
Bending stiffness of stent systems with crimped stent (circumferential average with standard deviation, BMS blue, DES red)

The measured bending stiffness of the stent delivery systems with mounted stents is shown in Fig. 5. For BMS, the bending stiffness ranged from 22.8 Nmm^2^ (PRO-Kinetic Energy) to 59.2 Nmm^2^ (Integrity) and for DES from 30.5 Nmm^2^ (Promus PREMIER) to 98.7 Nmm^2^ (Resolute Integrity). Pairwise comparison between BMS and DES has shown highly significant lower bending stiffness for the BIOTRONIK PRO-Kinetic Energy vs. Orsiro (*p* < 0.001) and the Medtronic Integrity vs. Resolute Integrity (*p* < 0.001). The Terumo Kaname BMS was stiffer than its DES counterpart Ultimaster (*p* = 0.049). For all other investigated stent families, the differences were not significant.

### Elastic recoil

For BMS, the recoil values ranged between 5.00 ± 0.31% (MULTI-LINK 8) and 5.87 ± 0.54% (REBEL/Monorail) of the diameter at nominal pressure. The range for DES was from 4.07 ± 0.22% (Orsiro) to 5.70 ± 0.26% (Resolute Integrity). The pairwise comparison of the BMS vs. DES stents with the same stent design provided significant differences only for the BIOTRONIK PRO-Kinetic Energy vs. Orsiro stents (*p* < 0.001).

### Bending stiffness of expanded stent

An overview of the bending stiffness of expanded stents is given in Table 2. Most flexible BMS were the REBEL/Monorail (3.6 ± 0.9 Nmm^2^) and Kaname (4.3 ± 0.3 Nmm^2^) stents; interestingly, the most flexible DES were in the same order of magnitude (Promus PREMIER: 4.0 ± 0.7 Nmm^2^; Ultimaster: 4.4 ± 0.3 Nmm^2^). The stiffest stents measured in completely expanded state were the Abbott MULTI-LINK 8 (9.6 ± 1.4 Nmm^2^) and XIENCE Xpedition (11.0 ± 1.4 Nmm^2^).

In case of expanded stents, no significant differences were observed between BMS and DES of the same manufacturer.

### Scaffolding

**Fig. 6:**
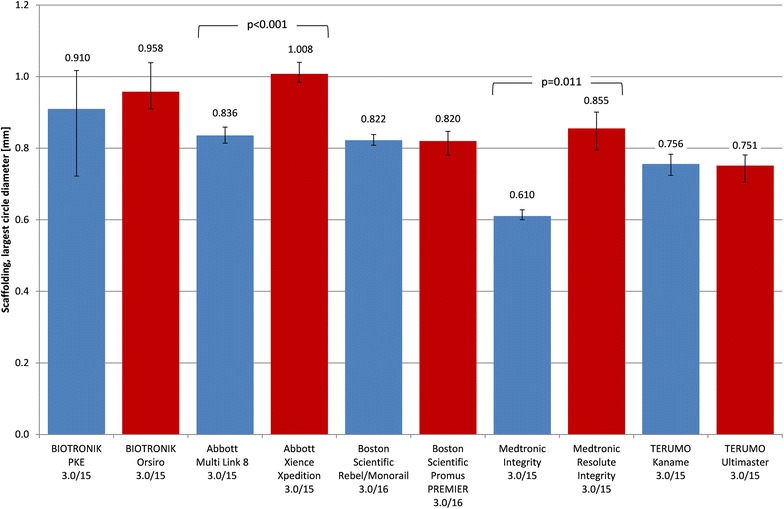
Diameter of the largest holes in the stent structure as a measure of scaffolding or side branch accessibility (mean ± min/max, BMS blue, DES red)

**Fig. 7:**
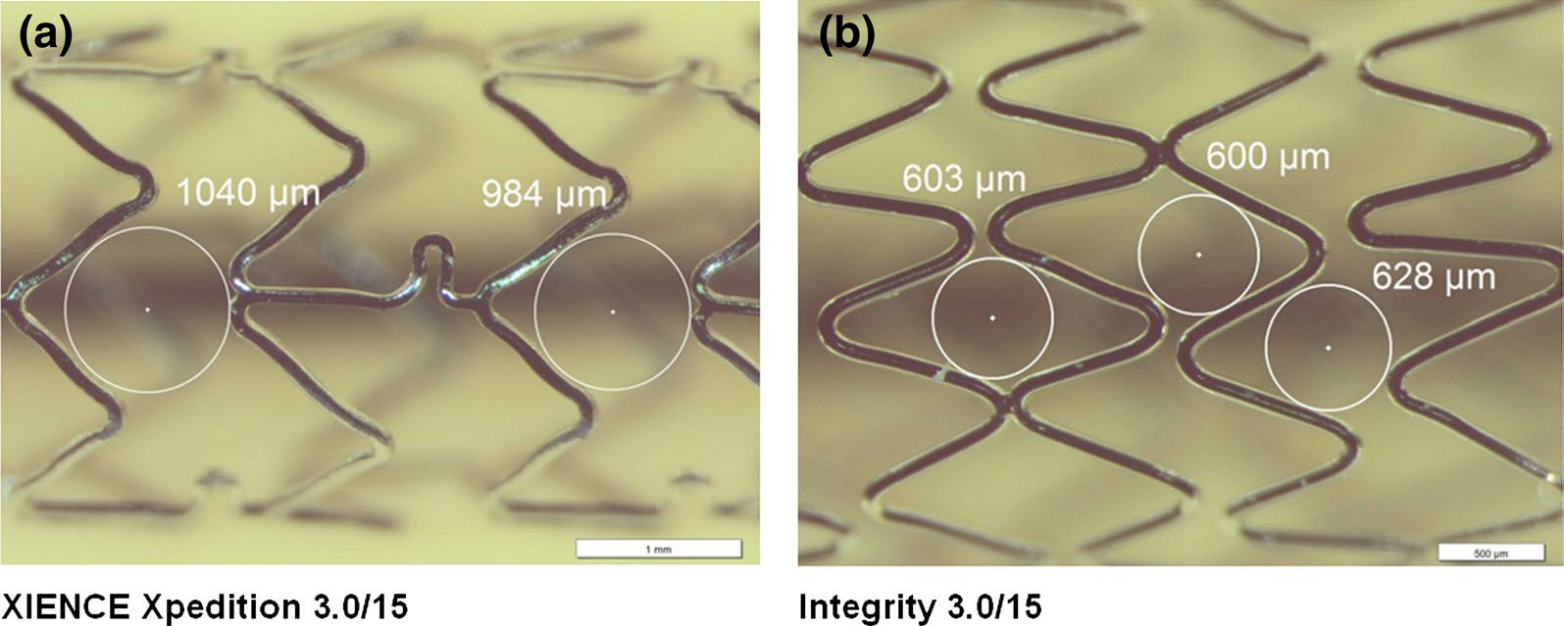
Scaffolding of stents expanded to nominal diameter of 3.0 mm—largest (**a**) and smallest (**b**) hole diameters

The potential for scaffolding is expressed by the diameter of the largest holes observed at the structure of expanded stents (Fig. 6). Overall, the smallest holes were measured at 0.610 mm (Integrity) and the largest with 1.008 mm (XIENCE Xpedition) (Fig. 7). Significant differences between BMS and DES of the same manufacturer were measured for the Abbott MULTI-LINK 8 vs. XIENCE Xpedition (*p* < 0.001) and the Medtronic Integrity vs. Resolute Integrity (*p* = 0.011) despite the fact of the same backbone design in each case. All other stent types showed no significant differences for this scaffolding parameter.

## Discussion

Mechanical properties are among the most critical aspects when judging the clinically relevant performance characteristics of coronary stents. Depending on the context of the individual target site, different properties might be required to meet the structural, geometric or morphologic challenge. Thus, for example, in ostial stenosis high radial forces may represent the key parameter while the bending stiffness might not be as critical. Conversely, in distal soft plaque lesions located in tortuous coronary arteries the opposite features are likely to be the case. Practically, each individual clinical scenario might require specific performance characteristics to achieve successful revascularization and, importantly, to minimize the procedural risk.

To date, the data on mechanical stent properties are rare in the literature. Consequently, the interventionists must rely on data provided by the industry and on their own experience. Thus, the stent selection is largely subjective and may depend on external not necessarily performance factors, such as pricing. Using the current generation of stents, technical success can be achieved in most cases. However, in technically demanding cases even minor differences in performance characteristics may be decisive in BMS, DES or BRS.

Measurements of mechanical properties of lesions and stents in vivo have been attempted, yet are difficult to perform and may not be practical at present despite of their relevance. While in vitro measurements might not accurately replicate the stent mechanical behaviour in vivo, they may provide reasonable estimates to support the operators’ decision making in selecting instrumentation. Particularly, in bail-out cases optimum selection of instrumentation is critical.

Based on empirical knowledge, BMS have been perceived to have better mechanical performance characteristics compared to DES and have been selected in critical cases. However, to our knowledge no data are available in the literature to support this perception.

In this study, we have performed head-to-head pairwise comparisons of BMS and DES of the same backbone design. The mechanical parameters studied were selected based on their potential impact on the technical success in complex sites such as high-grade lesions and tortuous vessels. The scarce data which are provided as part of the manufacturer’s specification were in good accordance with our measurements.

The importance of the full stent expansion, recoil and flexibility of stents has been emphasized in several studies [11, 13]. Biomechanical analyses were conducted using computational methods such as finite element analysis [22, 23]. For example, strut thickness was shown to be an important stent parameter with mechanical as well as fluid-mechanical implications [24] and stent length > 32 mm was found to be relevant for long-term clinical outcomes [25].

Published comparative data on mechanical parameters of coronary stent delivery systems and stents were limited largely to longitudinal stiffness [25]. The direct comparison of BMS and DES in [26] was based on the clinical data of BMS and first-generation DES based on paclitaxel and Sirolimus eluting stents, while stent design parameters were completely neglected.

In this study, direct comparison of BMS and DES of the same stent design revealed that the crimped profile of DES was bigger in two out of five tested pairs. With regard to crossability, three BMS types performed better compared to their DES counterpart and significant differences between manufacturers were measured. Bending stiffness of stent delivery systems varied largely between manufacturers and there was no consistent relationship between BMS and DES. Elastic recoil was not consistently different between BMS and DES and measured differences were manufacturer specific. Bending stiffness of expanded stents was not significantly greater for DES and scaffolding was consistently comparable or better with BMS.

Low forces while crossing the small stenosis model (ID = 1.1 mm) coincided with a lower profile of the PRO-Kinetic BMS vs. Orsiro DES. A similar correlation was found for the Integrity vs. Resolute Integrity stent systems (stenosis model ID = 1.2 mm). Significant lower cross forces were also measured for the REBEL/Monorail BMS compared to the Promus PREMIER DES system, even if the profile differences were not significantly different. In case of the XIENCE Xpedition, the crossability was better (passage of 1.1 mm) than that of the MULTI-LINK 8 BMS systems which could pass only 1.2 mm. The crimped profiles were not different. For another example, see the BMS REBEL/Monorail system with a profile of 1.05 ± 0.01 mm which provided significantly lower cross forces (0.098 ± 0.014 N) at the tip of the stent system during passage of the 1.1 mm stenosis than the DES Orsiro system with a profile of 1.02 ± 0.01 mm (0.142 ± 0.017 N, *p* = 0.004). In conclusion, the low profile of a stent system offers the opportunity to pass narrow lesions. This is an obvious basic requirement. But among those systems which have comparable profiles, additional parameters, such as catheter, catheter tip and balloon technology including coating, are assumed to affect crossability. In particular not only the profile of the balloon shoulders, but also the flexibility and refold characteristics of the balloon are assumed to be essential. It appears to be necessary to determine functional data, such as crossability, in experimental setups.

Our study considered the bending stiffness, where a low stiffness stands for a high flexibility. Evaluation of the resulting data, however, provides only a weak connection between profile and bending stiffness of the crimped stent. Indeed, lowest profile of the PRO-Kinetic correlates with lowest bending stiffness (profile 0.97 mm, bending stiffness 22.82 Nmm^2^), but even the largest profile determined at the Kaname BMS and Ultimaster DES systems (1.13 mm) allows a stiffness of only 36.44 or 33.35 Nmm^2^, respectively. It is assumed that in most cases the differences responsible for the varying stiffness values lie in the catheter design rather than in that of the crimped stent. This assumption is supported by the fact that expanded stents are much more flexible than the crimped stent systems.

The highest stiffness was observed at the Integrity (59.15 Nmm^2^) and Resolute Integrity (98.72 Nmm^2^) stent systems which have moderate profiles (1.07 mm and 1.09 mm), but largest strut thickness at both BMS and DES (90 µm). At comparable stent structures, lower strut thickness would cause higher flexibility. An effect of the coating of DES on bending stiffness of expanded stents is not apparent [5] since no significant differences were measured. An effect of the coating was not observed for most other mechanical properties, such as radial strength (support function), radial and longitudinal stiffness, and was not found for elastic recoil either (Table 2).

Comparison of bending stiffness with scaffolding, expressed by the largest circular diameter of openings in the expanded stent structure, provided no reliable relationships.

To determine the potential clinical utility of our in vitro data, we compared the results with the procedural success rates published in clinical studies.

However, quantitative comparison appeared difficult because the definitions of angiographic, procedural, technical and clinical success differ between studies [27–30]. Despite the inhomogeneous use, the reported success rates are commonly high and close to immaculate.

For the PRO-Kinetic Energy, a stent deployment success of 97.2% is reported from the PRO-Heal registry [27]. An all-comer study of the same device provides 99.8% angiographic success [28]. The Orsiro device was investigated in a real-world registry (BIOFLOW III) yielding clinical device success in 98.8% and procedure success in 98.2% of the patients [29]. A procedural success of 100% was reported from the first-in-man evaluation of the Integrity stent [30]. For the Kaname stent system delivery success, device success, and lesion success rates (per lesion) were 99.3, 99.3, and 100%, respectively [10]. The VISION registry conducted for MULTI-LINK 8 stent systems provided only 0.8% device malfunction [4] and the Omega Clinical trial (REBEL stent system) yield procedural success of 95.4% and technical success of 98.5% [5].

The reported high procedural success rates likely reflect the excellent technical and interventional skill standards, yet it appears likely that technical difficult cases might have been underrepresented in these trials. In addition, in particular the data on achieved technical quality of revascularization are not available.

## Limitations

In vitro measurements might not exactly reflect the clinical performance, but likely provide reasonable estimates useful in clinical decision making. The sample sizes were small, but sufficient for simple statistics because variations of technical in vitro parameters were low compared to those of complex patient specific in vivo characteristics. Selection of included BMS and DES was subjective but reflects the authors’ efforts towards providing a representative view on current instrumentation.

## Conclusions

Coronary stents represent a key component of the interventional instrumentation. Safe delivery of the stent to the target site and efficient therapy of the target stenosis are the key issues concerning all coronary interventions. While in the majority of cases mechanical properties of the stent might not be critical to achieve technical and procedural success; in more technically challenging cases the selection of the most appropriate instrumentation might be crucial. In this study, the key mechanical performance parameters of stents and stent delivery systems measured in vitro revealed that substantial differences between BMS and DES exist indeed; however, these differences are product dependent and in most cases not group specific. Overall, BMS on average perform slightly better compared to DES in terms of profile, crossability and bending stiffness of the crimped stent on its delivery system. However, the differences observed were typically subtle and their clinical significance is open to discussion. Due to the partly considerable differences between the products, the information provided by this study might assist the interventionist in material selection and decision making.

## Abbreviations

BMS: bare metal stents
BRS: bioresorbable scaffolds
DES: drug-eluting stents
IFU: instructions for use
L-605: cobalt chromium alloy
MP35 N: cobalt nickel base alloy
n: number of test samples
NP: nominal pressure
PCI: percutaneous coronary intervention
PtCr: platinum chromium alloy
RMS: root mean square
SDS: stent delivery systems
